# Dr. Bennet T. Welch

**Published:** 1911-12

**Authors:** 


DR. BENNET T. WELCH of Buffalo, died of typhoid fever, October 29, 1911. He was a graduate of the University of Buffalo, 1907.

